# A global dataset for the projected impacts of climate change on four major crops

**DOI:** 10.1038/s41597-022-01150-7

**Published:** 2022-02-16

**Authors:** Toshihiro Hasegawa, Hitomi Wakatsuki, Hui Ju, Shalika Vyas, Gerald C. Nelson, Aidan Farrell, Delphine Deryng, Francisco Meza, David Makowski

**Affiliations:** 1 Institute for Agro-Environmental Sciences, National Agricultural and Food Research Organization, Tsukuba, Ibaraki, 305-8604 Japan; 2 Institute of Environment and sustainable Development in Agriculture, Chinese Academy of Agricultural Sciences (IEDA,CAAS), Beijing, 100081 China; 3 Alliance of Bioversity International and International Center for Tropical Agriculture (CIAT), Nairobi, Kenya; 4 University of Illinois, Urbana, IL USA; 5 The University of the West Indies, St. Augustine, Trinidad; 6 IRI THESys, Humboldt-Universität zu Berlin, Berlin, 10099 Germany; 7 Pontificia Universidad Católica de Chile, Santiago, Chile; 8 Applied mathematics and computer science (MIA 518), INRAE AgroParisTech, Université Paris-Saclay, 75231 Paris, France

**Keywords:** Environmental impact, Heat

## Abstract

Reliable estimates of the impacts of climate change on crop production are critical for assessing the sustainability of food systems. Global, regional, and site-specific crop simulation studies have been conducted for nearly four decades, representing valuable sources of information for climate change impact assessments. However, the wealth of data produced by these studies has not been made publicly available. Here, we develop a global dataset by consolidating previously published meta-analyses and data collected through a new literature search covering recent crop simulations. The new global dataset builds on 8703 simulations from 202 studies published between 1984 and 2020. It contains projected yields of four major crops (maize, rice, soybean, and wheat) in 91 countries under major emission scenarios for the 21st century, with and without adaptation measures, along with geographical coordinates, current temperature and precipitation levels, projected temperature and precipitation changes. This dataset provides a solid basis for a quantitative assessment of the impacts of climate change on crop production and will facilitate the rapidly developing data-driven machine learning applications.

## Abstract (summary)

**Table:** 

Measurement(s)	relative yield change
Technology Type(s)	crop simulation model
Factor Type(s)	geographic location • current average temperature • current annual precipitation • future mid-point • climate scenario • temperature change • annual precipitation change • CO2 ppm
Sample Characteristic - Organism	Zea mays • Oryza sativa • Glycine max • Triticum aestivum
Sample Characteristic - Environment	climate change
Sample Characteristic - Location	global

Machine-accessible metadata file describing the reported data: 10.6084/m9.figshare.17427674

## Background & Summary

Climate change affects many processes of food systems directly and indirectly^1^, but the primary effects often appear in crop production. Projections of crop production under future climate change have been studied since the early 1980s. From the 1990s onward, researchers have used future climate data and crop simulation models to project the impacts of climate change on crop yields under various scenarios^2^. Since then, crop simulation models have been used in hundreds of studies to simulate yields for different crops under a range of climate scenarios and growing conditions^3^. The results have been periodically reviewed and assessed by national and international organisations, in particular by the Intergovernmental Panel on Climate Change (IPCC) Working Group II, which provides policy-relevant scientific evidence for the impacts of and adaptation to climate change^3^. Review studies covering the last five IPCC assessment cycles confirm that the overall effects are negative but vary significantly among regions^4,5^.

Before 2010, simulation studies were conducted mainly by individual research groups using different climate models, target years, spatial resolution with local management and cultivar conditions. Since 2010, however, significant efforts have been made to coordinate modelling studies through Agricultural Model Intercomparison and Improvement Project (AgMIP)^6^, which compares results from multiple crop models using standardised inputs. Early AgMIP activities have disentangled sources of uncertainties in crop yield projections and revealed that yield projections are variable among crop models and that model ensemble mean or median often works better than a single model^7–10^, underpinning the importance of datasets based on multiple crop models.

Data sets including crop model simulations produced by AgMIP were subjected to statistical analysis and the results were used to quantify the impacts of climate change on major crops^11,12^. A versatile tool to aggregate simulated results is already available for global gridded studies^13^ to facilitate access to the data. Besides these coordinated efforts, however, many simulation results are scattered and not readily available for meta-analysis. To deliver policy-relevant quantitative information, we need to develop a shared and well-documented database that can be used to assess the impacts of different climate and adaptation scenarios on crop yields.

Here, we have developed a global database for potential use for the IPCC Working Group II assessment, obtained through two methods. The first method draws on the dataset used in the meta-analysis of Aggarwal, *et al*.^5^, which includes studies considered in the previous five cycles of IPCC assessments^4,14^. The second method is based on a new literature search of studies published during the sixth IPCC assessment cycle (covering the period 2014–2020) reporting crop simulations produced for several contrasting climate change scenarios. The combined dataset covers all six cycles of the IPCC assessment and can serve as a solid basis for analyses from the sixth IPCC assessment onward.

The dataset contains the most relevant variables for evaluating climate change impacts on yields of maize, rice, soybean, and rice for the 21st century. They include geographical coordinates, crop species, CO_2_ emission scenarios, CO_2_ concentrations, current temperature and precipitation levels, local and global warming degrees, projected changes in precipitation, the relative changes in yield as a percentage of the baseline period obtained with or without CO_2_ effects, and with or without implementation of different types of adaptation options.

## Methods

### Data collection

**Fig. 1:**
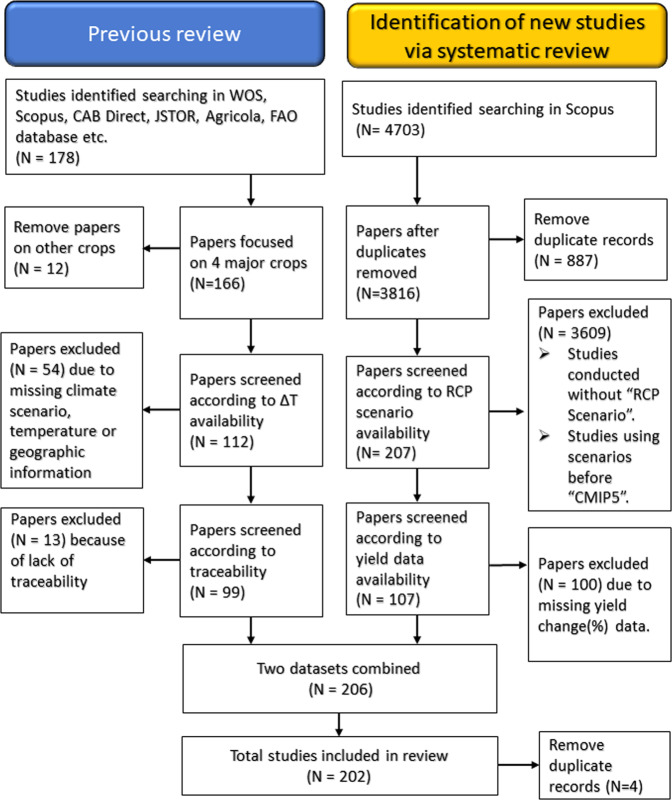
A diagram depicting paper collection and selection using the two search strategies. N is the number of studies.

As shown in a PRISMA diagram (Fig. 1), we obtained data through two methods to develop this dataset. The first method is based on the previous meta-analysis by Aggarwal *et al*.^5^, which includes studies published before 2016 (Aggarwal-DS, hereafter). This meta-analysis builds on the dataset used for the 5^th^ IPCC assessment report^4,14^ and an additional search through three types of databases: Scientific database (Scopus, Web of Science, CAB Direct, JSTOR, Agricola etc), journals and open access repositories, and institutional Websites (FAO database, AgMIP Database, World Bank, etc.) and Google Scholar. See Aggarwal *et al*.^5^ for details. Briefly, the search terms used by Aggarwal *et al*.^5^ include “agriculture” or “crop “or “farm” or “crop yield” or “crop yields” or “farm yields” or “crop productivity” or “agricultural productivity” or “maize” or “rice” or “wheat” and “climate change assessment” or “climate impacts” or “impact assessments” or “climate change impact” or “climate impact” or “effect of climate” or “impact of climate change”. The number of selected papers covering the four major crops is 166. We further screened them according to the availability of local temperature rise and geographical information, and traceability, resulting in 99 studies published between 1984 and 2016.

The second method relies on a new recent literature review conducted using Scopus in March 2020 for four major crops (maize, rice, soybean, and wheat) for peer-review papers published from 2014 onward in line with the sixth assessment cycle of IPCC. In this method, we used several combinations of terms to retrieve relevant studies reporting simulations of the impacts of climate change on crop yields using recent climate change scenarios.

For maize, the following search equation was used: PUBYEAR > 2013 AND TITLE-ABS-KEY((maize OR corn) AND ((“greenhouse gas” OR “global warming” OR “climate change” OR “climate variability” OR “climate warming”)) AND NOT (emissions OR mitigation OR REDD OR MRV)).

Similar search equations were used for the other crops. Collectively, this search returned a total of 4703 references between 2014 and 2020: 1899 for maize, 1790 for wheat, 757 for rice, and 257 for soybean with some duplications because some papers studied multiple crops. Removing the duplicates, the number is down to 3816 studies.

To collect climate-scenario-based simulations, we then selected a subset of studies including the following terms related to climate scenarios in titles, abstracts, or authors’ keywords; “RCP”, “RCP2.6”, “RCP6.0”, “RCP4.5”, “RCP8.5”, “CMIP5”, and “CMIP6”. RCP stands for the Representative Concentration Pathways^15^, and each RCP corresponds to a greenhouse gas concentration trajectory describing different future greenhouse gas emission levels. The number followed by RCP is the level of radiative forcing (Wm^−2^) reached at the end of the 21^st^ century, which increases with the volume of greenhouse gas emitted to the atmosphere^16^. CMIP5^17^ and CMIP6^18^ are the Coupled Model Intercomparison Project Phase 5 and Phase 6, respectively, where groups of different earth system models (ESMs) provide global-scale climate projections based on different RCPs. Additionally, “process-based model” was used to search in the authors’ keywords to select for studies that use crop simulation models under CMIP5 or CMIP6 climate scenarios. As of March 2020, no results were found for CMIP6 in any search results.

This screening process resulted in a total of 207 references all together for four major crops. These studies were further evaluated for their variables and data availability; studies not reporting yield data were excluded. Projected yields with and without adaptations and yields of the baseline period were extracted, along with geographical coordinates, crop species, greenhouse gas emission scenarios, and adaptation options. We also tried to obtain local and global temperature changes and CO_2_ concentrations as much as possible. In addition to extracting data from the literature, we contacted several authors of grid simulation studies to provide aggregated results for countries or regions. The authors of the three grid simulation studies responded and provided baseline and projected yields, annual temperature and precipitation data aggregated over for countries or regions^19–21^. The results from different ESMs were then averaged.

We removed duplicates between the datasets produced by the two methods and ultimatelly obtained a total of 202 unique studies. Both datasets include studies with different spatial scales: site-based, regional, and global. Among these, the results from the global gridded crop models were aggregated to country levels, and we focused on top-producing countries, which account for 95% of the world’s production of each commodity as of 2010 (FAOSTAT, http://www.fao.org/faostat/en/, accessed on September 4, 2020). As a result, the dataset contains 8,703 sets of yield projections during the 21^st^ century from studies published between 1984 and 2020 (Online-only Table 1).

### Relative yield impacts

Simulated grain mass per unit land area is used to derive the impact of climate change on yield (YI), which is defined as:

$${\rm{YI}}\left( \% \right)=\left({{\rm{Y}}}_{{\rm{f}}}/{{\rm{Y}}}_{{\rm{b}}}-{\rm{1}}\right)\times {\rm{100}}$$

Where Y_f_ is the future yield, and Y_b_ is the baseline yield. One study^20^ simulated yields separately under both climate change and counterfactual non-climate change scenarios from the pre-industrial era toward the end of the 21^st^ century, also accounting for yield increases due to non-climatic technological factors over time. In this case, YI obtained with the above equation under the climate change scenario was not fully relevant because it combines effects of both climate change and technological factors. Thus, for this study, YI was derived from the average yield in the 2001–2010 period under climate change and the average yield in the same period assuming no climate change, as follows:

$${\rm{Y}}{\rm{I}}({\rm{ \% }})=[\{({{\rm{Y}}}_{{\rm{f}}{\rm{\_}}{\rm{c}}{\rm{c}}}-{{\rm{Y}}}_{{\rm{b}}{\rm{\_}}{\rm{c}}{\rm{c}}})-({{\rm{Y}}}_{{\rm{f}}{\rm{\_}}{\rm{n}}{\rm{c}}{\rm{c}}}-{{\rm{Y}}}_{{\rm{b}}{\rm{\_}}{\rm{n}}{\rm{c}}{\rm{c}}})\}/{{\rm{Y}}}_{{\rm{b}}{\rm{\_}}{\rm{c}}{\rm{c}}}-1]\times 100$$

Where Y_f_cc_ and Y_b_cc_ are the future and baseline average yields with climate change, Y_f_ncc_ and Y_b_ncc_ are the future and baseline average yields under counterfactual no climate change scenario.

Projected absolute grain yield (t/ha) is also included in the dataset, when available. These values should be used with caution because absolute grains yields are not always comparable due to the use of different yield definitions or assumptions. Different definitions include graded or non-graded yields, husked or unhusked, milled or non-milled yield. Moisture content correction factors can also be different, but these are not often explicitly indicated in the literature. Contrary to absolute yields, relative yields are unitless and rule out differences of yield defintions between studies.

### Adaptation to climate changes

Various management or cultivar options are tested in the simulations. If the authors of the article consider these options as ways to adapt crops to climate change, we treat them as adaptation options, which are categorised into fertiliser, irrigation, cultivar, soil organic matter management, planting time, tillage, and others. Specifically, in the fertiliser option, if the amount and timing of fertiliser application are changed from the current conventional method, we treat them as adaptation. In the irrigation option, if the simulation program determines the irrigation scheduling based on the crop growth, climatic and soil moisture conditions, we treat this as adaptation because the management is adjusted to future climatic conditions. If rainfed and irrigated conditions are simulated separately, we do not consider irrigation as an adaptation. We define cultivar option as the use of cultivars of different maturity groups and/or higher heat tolerance than conventional cultivars. The planting time option corresponds to a shift of planting time from conventional timing. If multiple planting times are tested, we select the one that gives the best yield. The soil organic matter management option corresponds to application of compost and/or crop residue. The tillage option corresponds to reduced- or no-till cultivation compared to no conventional tillage. When studies consider adaptation options, we compute YI from the ratio of yield with adaptation under climate change to baseline yield without adaptation. To measure our capacity to adapt to climate change, we calculated adaptation potential - defined as the difference between yield impacts with and without adaptation - when a pair of yield values were available in the same study.

### Temperature and precipitation changes

Both local temperature rise (ΔT_l_) and global mean temperature rise (ΔT_g_) from the baseline period have important implications. The former directly affects crop growth and yield, and the latter represents a global target associated with mitigation activities. We extracted both ΔT_l_ and ΔT_g_ from the literature as much as possible, but ΔT_g_ is not available in many studies. In such cases, we estimated ΔT_g_ using the Warming Attribution Calculator (http://wlcalc.climateanalytics.org/choices). In the dataset, we provide two estimates for ΔT_g_: one from the current baseline period (2001–2010) and the other from the preindustrial era (1850–1900). We also extracted precipitation changes (ΔPr) and baseline precipitation data reported in the selected studies. When only relative changes were available for precipitation data, we estimated ΔPr using the reported relative change and current precipitation levels described in the next section.

### Current temperature and precipitation levels

Current annual mean temperatures and precipitation were obtained from the W5E5 dataset^22^, which was compiled to support the bias adjustment of climate input data for the impact assessments performed in Phase 3b of the Inter-Sectoral Impact Model Intercomparison Project (ISIMIP3b, https://www.isimip.org/protocol/3/). The W5E5 dataset includes half-degree grid resolution daily mean temperature and precipitation data from 1979 to 2016, which we averaged for the period from 2001 and 2010. They were then extracted for each simulation site or region using the geographic information. For global simulations, which were aggregated to the country level, central coordinates were used to link gridded temperature and precipitation data with each country. As centroids may not represent the centre of the growing regions, particularly in large countries, growing-area weighted averages of annual temperature and precipitation were also provided using MIRCA 2000^23^, which contains half-degree grid harvested areas (a total of irrigated and rainfed) around the year 2000.

### CO_2_ concentrations

Several studies report two series of yield simulations obtained using two CO_2_ levels to infer the CO_2_ fertilization effects: one obtained with CO_2_ concentrations fixed at the current levels and the other obtained with increased future CO_2_ concentrations provided by the emission scenario considered. In the dataset, we make this explicit in the following two variables:

1. CO_2_: Binary variable equal to “Yes” if future CO_2_ concentrations from the emission scenarios were used and “No” if the current CO_2_ concentration was used for the yield simulations.
2. CO_2_ ppm: if available, CO_2_ concentration was extracted from the original paper. If not, we calculated it from projected changes in CO_2_ concentrations based on the scenarios and periods studied. CO_2_ concentration data were obtained from https://www.ipcc-data.org/observ/ddc_co2.html for CMIP3 and Meinshausen, *et al*.^16^ (http://www.pik-potsdam.de/~mmalte/rcps/) for CMIP5.

### Baseline correction

Because baseline periods differed between studies, we corrected YI, ΔT_l_, ΔT_g,_ ΔPr to the 2001–2010 baseline period by a linear interpolation method following Aggarwal *et al*.^5^. Namely, the impacts YI were first divided by the year gap between the future period midpoint year and the baseline period midpoint year of the original study. The impact per year was then multiplied by the year gap from our reference baseline period midpoint year (2005). The same method was applied to express ΔT_l_ and ΔPr relatively to 2001–2010.

We made all data publicly available to increase accessibility (see Data Records section for access).

## Data Records

**Fig. 2:**
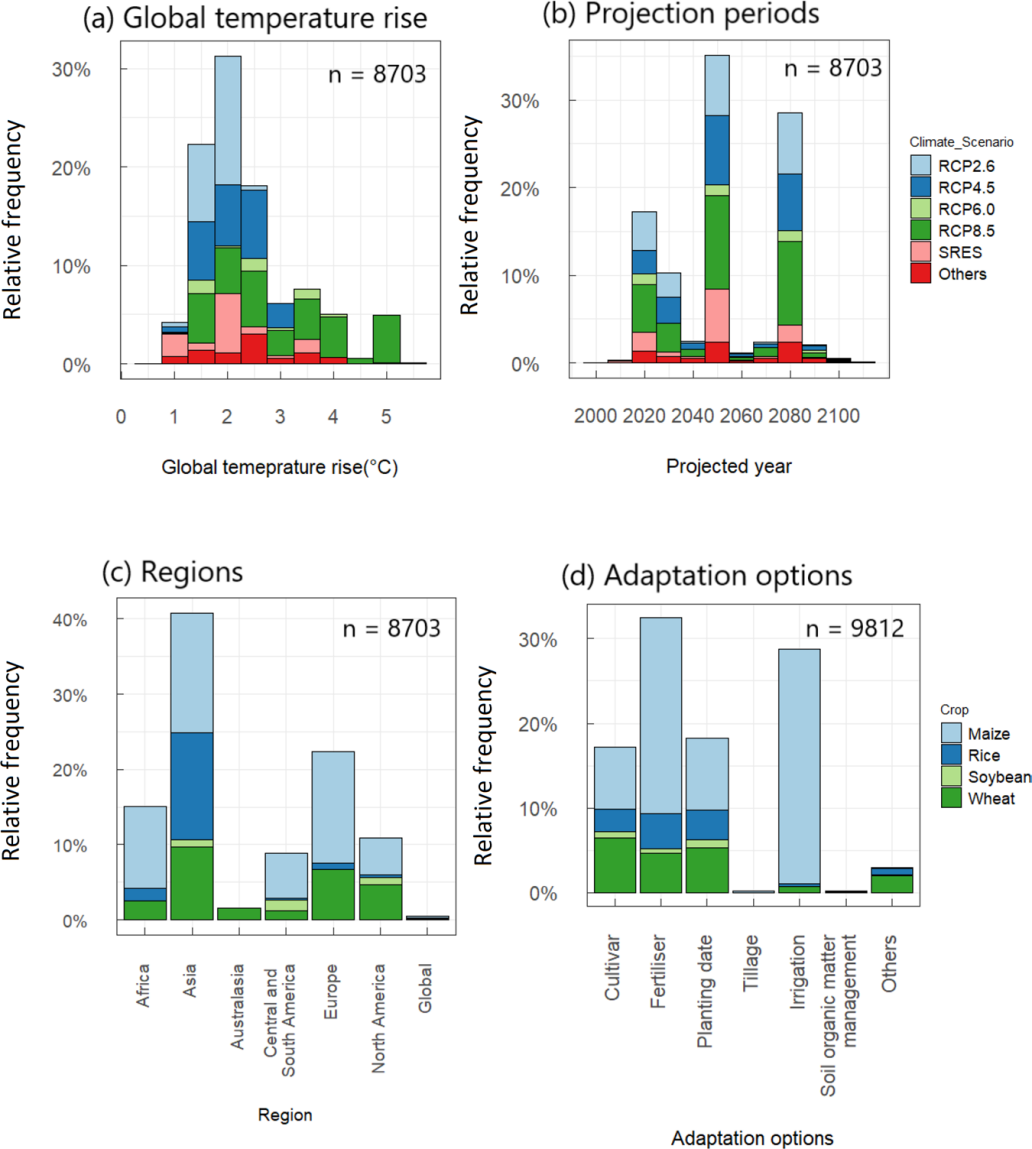
Data availability of crop yield simulations and its breakdown. **(a)** By global temperature rise from the preindustrial era and climate scenarios, **(b)** By projected time periods (midpoint years) and climate scenarios, **(c)** IPCC regions^29^ and crop species, and **(d)** adaptation options and crop species. Note that n = 9812 in adaptation options **(d)** exceeds the total number of simulations (8703) because we collectively add all the options used in the simulations, including those that use multiple options. n is the number of simulation results.

**Fig. 3:**
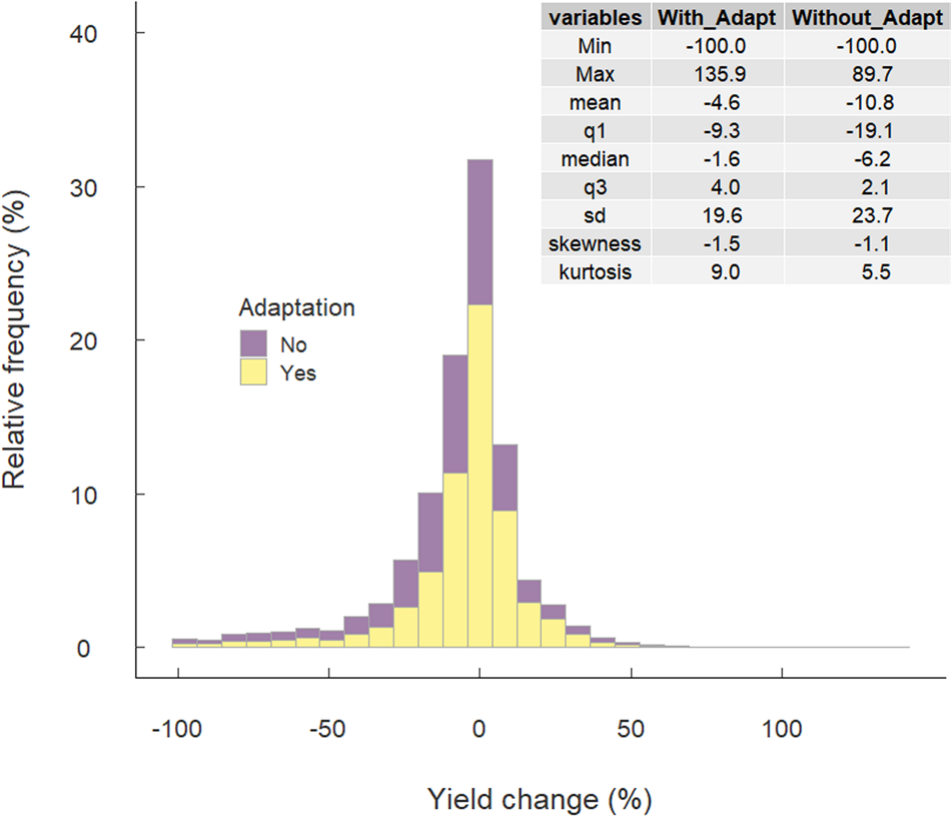
Distribution of relative yield change due to climate change from the baseline period (2001–2010) with and without adaptation.

**Fig. 4:**
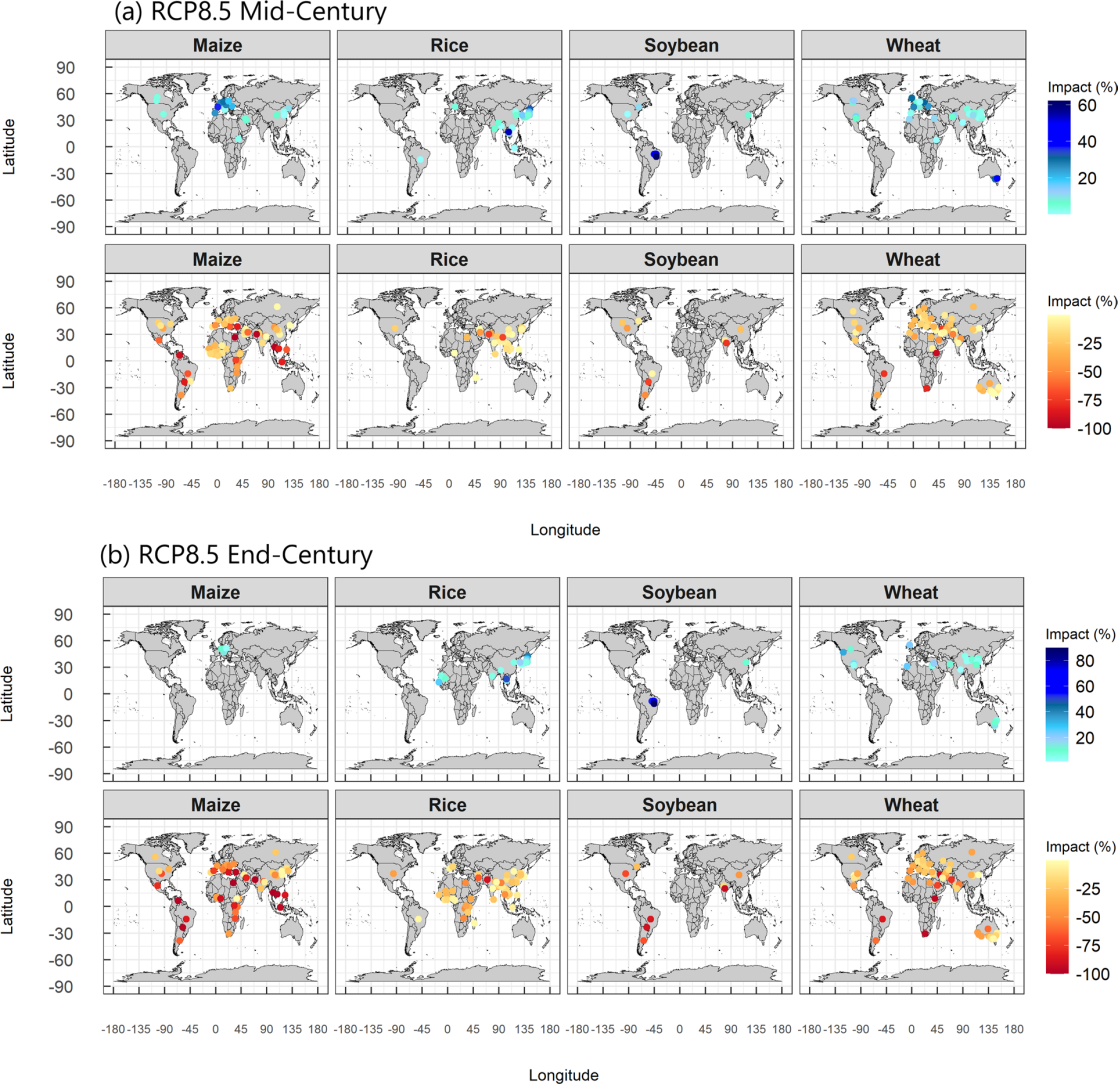
Climate change impacts (% of yield change from the baseline period) on four crops without adaptation under RCP8.5. (**a**) Mid-century; (**b**) End-Century. Maps with bluish symbols show positive effects (yield gain); Maps with reddish symbols show negative effects (yield loss). Projections under RCP2.6 and RCP4.5 are given in Supplementary Fig. S3.

**Fig. 5:**
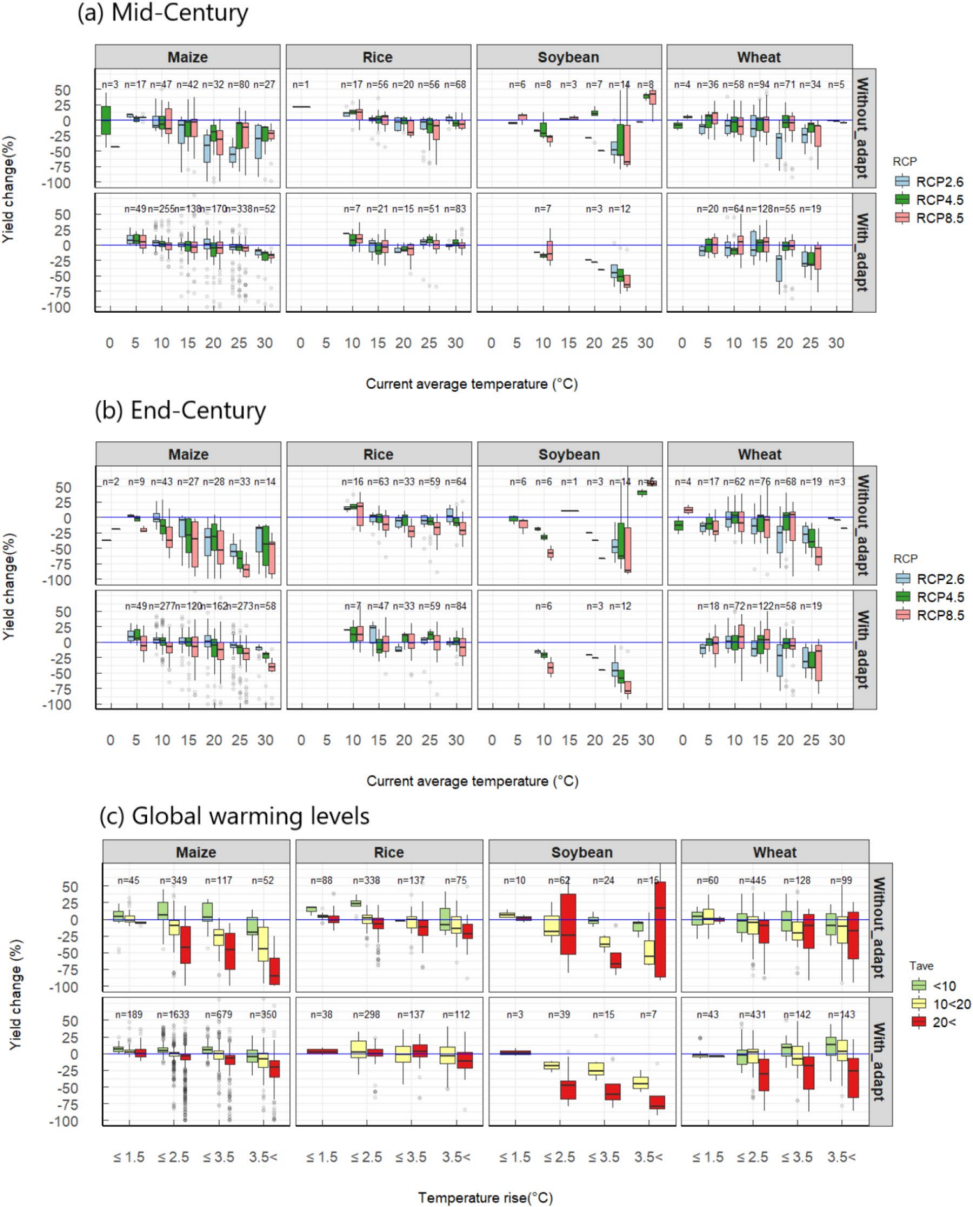
Projected yield changes relative to the baseline period (2001–2010). **(a)** Mid-century (MC) projections without adaptation under RCP8.5 scenario, upper panels showing positive impacts and lower panels negative impacts, **(b)** End-century (EC) projections under three RCP scenarios by current annual temperature (T_ave_), and **(c)** Yield change as a function of global temperature rise from the pre-industrial period by three T_ave_ levels. The box is the interquartile range (IQR) and the middle line in the box represents the median. The upper- and lower-end of whiskers are median 1.5 × IQR ± median. Open circles are values outside the 1.5 × IQR.

**Fig. 6:**
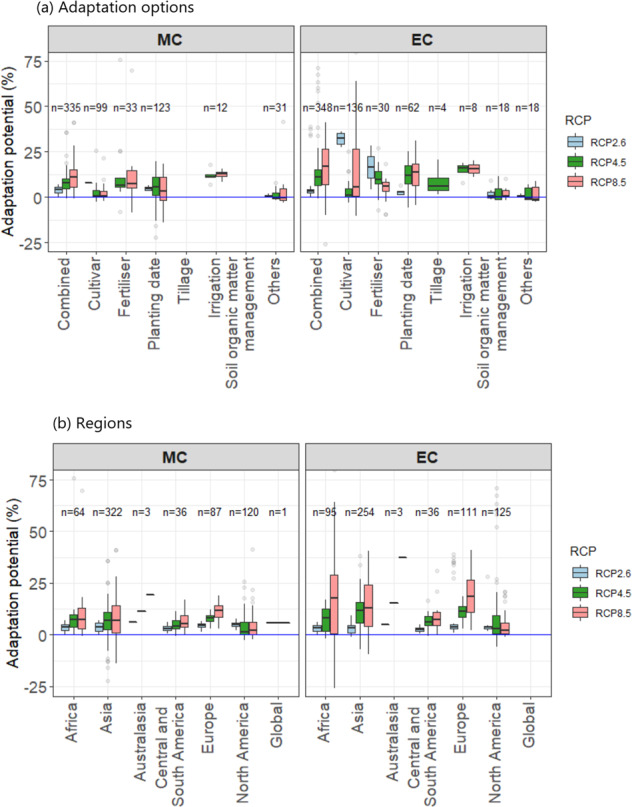
Adaptation potential, defined as the difference between yield impacts with and without adaptation in projected yield impacts, for three RCPs by mid- and end-century (MC, EC). The box is the interquartile range (IQR) and the middle line in the box represents the median. The upper- and lower-end of whiskers are median 1.5 × IQR ± median. Open circles are values outside the 1.5 × IQR. **(a)** By adaptation options and **(b)** by IPCC regions.

All the data and R scripsts associated with the dataset are stored in the figshare repository^24^, where the following files are uploaded:

1. “Projected_impacts_datasheet_11.24.2021.xlsx” includes three worksheets. “Projected_impacts” worksheet contains the final dataset after screening, and “Adaptation_potential” is the extracted subset of the paired data comparing yield impacts with and without adaptation. “Excluded” has untraceable simulation results in the Aggarwal-DS.
2. “Meta-data_11.25.2021.xlsx” contains the summary of the dataset, such as the definition and unit of the variables used in “Projected_impacts_datatasheet.xlsx”.
3. “Online_only_summary_tables_11.18.2021.xlsx” contains data distribution, median, and mean impacts of climate change, presented in the online-only tables.
4. “Supplementary_materials_11.29.2021.pdf” contains methods for estimating local temperature rise and summary distribution of climate change impacts on four crop yields.
5. “Reference_11.24.2021.docx” provides a list of references that provided data.
6. “R_script_for_Hasegawa_et.al.11.26.2021.zip” contains R scripts used to estimate missing values of ΔT_l_,ΔT_l_ and ΔPr and draw Figs. 2–6.

### Coverage of the data

A total of 8703 yield simulations are registered in the consolidated dataset. The number of simulations grows exponentially with publication year: 20 in the 1980s, 304 in 1990s, 830 in 2000s and 7549 in 2010s (Online-only Table 1). About 80% of the simulations use CMIP5 climate scenarios, and 11% use CMIP3. From CMIP5, RCP2.6, RCP4.5 and RCP8.5 are the most used concentration pathways (Online-only Table 2, Fig. 2a). ΔT_g_ from the baseline period (2001–2010) ranges from 0 to 4.8 °C (0.8 to 5.6 °C from the preindustrial period). Almost all simulations with ΔT_g_ > 3 °C use RCP8.5, resulting in a greater ΔTg range under CMIP5 (RCPs) than under previous scenarios (SRES and others).

Projected time periods span widely in the 21^st^ century, but the midpoint years peak at 2020 for the near future, 2050 for mid-century, and 2080 for end-century (Fig. 2b). Major emission scenarios such as RCP2.6, 4.5 and 8.5 are almost equally distributed across time periods. About 5% of the simulations assume no CO_2_ fertilisation effects.

Relative frequency of the regions studied generally reflects harvested areas of the four crops in each region (Fig. 2c). About 41% of the simulations were performed in Asia, which accounts for about 47% of the harvested area of the four major crops (mean of 2017–2019, FAOSTAT, http://www.fao.org/faostat/en/, accessed on April 28, 2021). Europe is slightly overstudied (22%) for its world share of the harvested areas (12%). Central and South Americas is slightly under-researched (9%) for the regional share of harvested areas (15%), whereas Africa’s share (15%) is comparable to the area harvested (10%). Altogether global harvested areas for these four major crops is 7 × 10^8^ ha: wheat represents 31% of this area, followed by maize (28%), rice (23%) and soybean (18%). Maize studies are over represented, accounting for about half of the simulations (52%), followed by wheat (26%) and rice (17%); soybean accounts only for 3% of the simulations (Fig. 2c). Regionally, maize and wheat are harvested across almost all regions, and simulations follow the actual distribution of these crops. Rice is predominantly studied in Asia, reflecting actual distribution (85% of the harvested area is in Asia). Soybean remains understudied compared to the other three crops despite its large cultivated area (about 75% of the rice harvested area). Regionally, simulation sites or regions for soybean are mostly in the Americas, which account for 76% of the total soybean harvested area.

About 39% of the simulations (3376) use current management practices, and the rest (5327) consider different management or cultivars as adaptation options (Fig. 1d). More than half of the simulations are run with multiple options. Among these options, fertiliser accounts for 32% followed by irrigation (29%), cultivar and planting date (17% each). There are 2005 pairs of yield simulations available for comparing results obtained with and without adaptation. These pairs of yield data can be used to compute the adaptation potentials of the different options considered.

## Technical Validation

### Data quality check

We repeatedly checked the data with multiple authors for the new dataset. For the Aggarwal-DS, we reviewed the sources of references. In case of missing information such as climate scenarios, CO_2_ concentration, or temperature increase, we came back to the original reference. Inconsistencies between the dataset and original papers were corrected when possible. Overall, corrections were made on 333 simulations from 10 studies, which we flag with “*” in the remark column of the dataset. We removed all data of the Aggarwal-DS that were untraceable in the original paper. This quality control excluded 47 simulations from 9 articles listed in the “Excluded” sheet.

We first examined the distribution of the climate change impacts on crop yields, which span from −100 to 136% (Fig. 3). This distribution is skewed to the left, as indicated by the negative skewness. The large kurtosis shows that distribution tails are longer than than those of the normal distribution. We tested the effects of potential outliers outside the 1.5-fold interquartile range (IQR) on the summary statistics of the climate change impacts on crop yields^25^. Removing values outside the 1.5-fold IQR decreases the number of simulations by 907(10.4%) and the negative effects of climate change on crop yields by 3.0% for the mean and 0.6% for the median, suggesting that the deletion affected the original distribution. We, therefore, keep all the simulation results in the dataset.

### Methods to estimate local temperature and precipitation changes

Out of 8703 simulations, local temperature change (ΔT_l_) and global temperature range (ΔT_g_) were available in 4316 and 8109 simulations, respectively. To estimate ΔT_l_ for 3793 simulations with missing ΔT_l_, we examined the relationship between ΔT_l_ and the following six input variables in 4316 simulations where ΔT_l_ was available: ΔT_g_, average temperature (area weighted), latitudes, longitudes, time periods, and emission scenarios. Values of ΔT_l_ were estimated using random forest algorithms trained to establish a function relating local temperature rise to the six inputs considered. We tested and compared four models based on different combinations of the input variables. Among the four models, a reduced model with three variables (ΔT_g_, latitude, and longitude) showed the highest percentage of explained variance (97.1%), and led to a cross-validated RMSE as low as 0.18 °C (Supplementary Table S1 and Fig. S1). We, therefore, used the reduced model to impute ΔT_l_ for the 4430 missing data. We also estimated ΔT_g_ for 504 simulations with missing ΔT_l_ from ΔT_g_, average temperature (area weighted), latitude, longitude, climate scenarios, future-midpoint year (Supplementary Table S2 and Fig. S2).

Likewise, we applied a random forest model to estimate ΔPr from current annual precipitation and average temperature (area weighted), latitude, longitude, local temperature change from 2005), climate scenario, future mid-point year, and climate change impact on yield relatively to 2005. Among eight models tested, a one with ΔT_g_, ΔT_l_, latitude, longitude, RCP, future-mid-point year and current annual precipitation perfomed best, which accounted for 96.9% of the out-of-bag variation of the data (n = 3560) and led to a cross-validated RMSE was 18 mm (Supplementary Table S3). We then applied this model to estimate all missing ΔPr.

### Comparison with previous studies

The overall effects of climate change on crop yields are negative, with the mean and median of −11% and −6.2% without adaptation and −4.6% and −1.6% with adaptation, respectively (Online-only Tables 3 and 4). The median per-decade yield impact without adaptation is −2.1% for maize, −1.2% for soybean, −0.7% for rice, and −1.2% for wheat (Table 1), which are consistent with previous IPCC assessments^14^. The median per-warming-degree impact is −7.1% for maize, −4.0% for soybean, −2.3% for rice, and −3.7% for wheat (Table 1). Per-degree yield impacts for each crop are generally within the range reported in the previous meta-analysis^11^. Among the four crops, soybean has the least number of simulations, resulting in a greater variation in both per-decade and per-degree impacts. Maize consistently shows the largest negative impacts, while rice shows the least.

**Table 1:** Summary statistics of climate change impacts on four major crops expresses as per decade impact and per degree impact without adaptation.

	Per decade impact (% decade^−1^)				Per degree impact (% °C^−1^)			
	Maize	Rice	Soybean	Wheat	Maize	Rice	Soybean	Wheat
Minimum	−40.0	−40.8	−30.0	−35.4	−158.7	−71.7	−112.6	−122.3
Maximum	14.2	26.2	13.8	21.2	70.8	120.7	58.3	153.7
Mean	−3.9	−1.4	−2.6	−1.8	−13.5	−2.6	−8.8	−5.6
1st quartile	−5.5	−2.5	−6.7	−3.5	−18.1	−7.1	−16.9	−10.9
Median	−2.1	−0.7	−1.2	−1.2	−7.1	−2.3	−4.0	−3.7
3rd quartile	−0.3	0.8	1.7	0.7	−1.1	2.7	6.3	2.3
Standard deviation	7.0	4.7	7.4	5.0	25.4	12.0	26.3	17.4
Skewness	−1.8	−2.6	−0.9	−1.7	−1.9	1.0	−1.1	−1.2
Kurtosis	8.6	21.9	4.7	10.8	8.6	24.4	5.2	17.4

The climate change impacts by IPCC regional groups reveals that Europe and North America are expected to be less affected by climate change in the mid-century (MC) and the end-century (EC) than Africa, Central and South America, particularly for maize and soybean. Both positive and negative effects are mixed in all regions (Fig. 4, Supplementary Figs. S3, S4).

Regional differences in the impacts in MC and EC are associated with the current temperature level. In MC, positive or neutral effects are projected when current annual average temperatures (T_ave_) are below 10–15 °C, but the effects become negative as T_ave_ increases beyond these levels regardless of RCPs (Fig. 5a). This accounts for the regional differences as a function of latitude reported in previous meta-analyses^4,5^. In EC, the threshold T_ave_ shifts lower, and the negative effects become more severe, particularly under a high emission scenario (RCP8.5) (Fig. 5b). The effect of ΔT_g_ from the baseline period onYI differs depending on the T_ave_ (Fig. 5c); At T_ave_ < 10 °C, YI is generally neutral even where ΔT_g_ > 2 °C in most crops, but at T_ave_ > 20 °C, YI is negative even with small ΔT_g,_ notably in maize. The difference in the YI dependence on ΔT_g_ between regions is also consistent with the previous study^4^.

Adaptation potential averaged 7.3% in MC and 11.6% in EC (Fig. 6, Supplementary Fig. S5), which is not sufficient to offset the negative impacts, particularly in currently warmer regions. Residual damages will thus likely remain even with adaptation, which is also supported by other lines of evidence^26,27^.

## Usage Notes

Crop yield simulation studies can provide a narrative of when, where, and what will happen to crop production under different GHG emissions and climate scenarios. They are also expected to provide quantitative information on the potential and limits to adaptation. However, robust estimates covering different temporal and spatial scales need to draw on multiple results obtained from various simulation studies. Nearly four decades have passed since the model projections based on future climate scenarios started. This dataset covers the entire period of simulation studies using climate scenarios, which can help update the quantitative review of climate change impacts on crops. The full list of references is provided in the reference file (10.6084/m9.figshare.14691579.v4).

Currently, studies are heavily biased towards major cereals such as maize, rice, and wheat, but this can be expanded to include other crops. As of 2020, our literature search failed to find published reports using CMIP6 climate scenarios, but this dataset can be easily updated when new simulations using new climate scenarios or other crop species become available. The next IPCC assessment cycle can fully utilise this dataset by adding the latest simulation results.

One of the caveats to the current dataset is that it only includes crop yield data, notwithstanding crop simulation studies are expected to produce other results than yield. Because of the recent progress in crop modelling, grain quality projections are emerging^28^. We have extensively included the temperature and precipitation levels to account for the impacts concerning the warming and current temperature, but there is a need to include other key climatic variables such as soil moisture. It will be useful to expand our dataset in the future to include this type of data.

## Supplementary information

Supplementary materials

## Data Availability

Script files were created using the R statistical programming to estimate missing values of ΔT_l_, ΔT_l_ and ΔPr and draw Figs. 2–6 which are available in the figshare repository^24^.

## Online-only Tables

**Online-only Table 1:** Number of simulations by publication years.

Publication year	N	Year	N
1984	1	1984–1990	20
1986	1	1991–2000	304
1987	2	2001–2010	830
1988	8	2011–2020	7549
1989	1		
1990	7		
1991	2		
1992	1		
1993	26		
1994	49		
1995	24		
1996	33		
1997	28		
1998	16		
1999	79		
2000	46		
2001	44		
2002	69		
2003	90		
2004	321		
2005	74		
2006	12		
2007	53		
2008	62		
2009	35		
2010	70		
2011	435		
2012	18		
2013	12		
2014	2884		
2015	106		
2016	231		
2017	2184		
2018	835		
2019	555		
2020	289		

**Online-only Table 2:** Number of simulations and its breakdown by crops, region, climate scenarios, timeframes and adaptation options.

Crop	Region	RCP2.6			RCP4.5			RCP6.0			RCP8.5			SRES			Others			Sub total	Total
		NF	MC	EC	NF	MC	EC	NF	MC	EC	NF	MC	EC	NF	MC	EC	NF	MC	EC		
Maize	Africa	92	89	92	49	61	52	21	21	21	87	108	90	46	52	23	18	12	17	951	4589
	Asia	121	123	128	71	128	73	30	31	31	109	174	117	66	105	48	4	5	14	1378	
	Central and South America	56	56	57	26	30	27	11	11	12	49	53	50	9	25	5	7	28	6	518	
	Europe	147	147	147	78	78	70	30	30	30	137	137	129	11	67	11	19	10	18	1296	
	North America	28	25	22	11	25	31	4	6	22	21	42	25	12	18	5	45	54	33	429	
	Global			1		1	1			1	5	5	3							17	
Rice	Africa	6	6	6	6	6	6				12	12	68	3	3	3		6		143	1519
	Asia	51	60	55	108	145	131	8	12	8	137	148	153	49	45	39	13	13	65	1240	
	Central and South America	2	2	2	2	2	2				4	4	4	2	1	1				28	
	Europe	16	2	18		2	2				16	2	18							76	
	North America	2	2	2	2	2	2				4	4	4	1	1	1				27	
	Global			1	1		1			1			1							5	
Soybean	Asia	6	6	6	6	7	6				9	8	9	1	8		4	4	4	84	297
	Central and South America	6	7	6	9	13	10				6	11	10	1	12		6	21	6	124	
	North America	6	3	2	2	9	5				2	8	5	1	6	1	15	2	17	84	
	Global			1		1	1			1			1							5	
Wheat	Africa	12	12	12	12	18	18				22	29	34	4	24	4		10	8	219	2298
	Asia	27	43	41	64	115	91	2	18	10	66	127	99	24	56	19	15	2	26	845	
	Australasia	2	2	2	2	14	22				3	15	23	1	5	9	1	27	9	137	
	Central and South America	6	6	6	6	7	6				9	10	9	5	15	3	2	11	2	103	
	Europe	32	32	32	40	42	34				57	60	51	22	81	16	12	22	46	579	
	North America	6	4	6	5	61	62			4	9	68	62	2	23	3	21	39	30	405	
	Global		1	1		1	2			1		3	1							10	
Sub total		624	628	646	500	768	655	106	129	142	764	1028	966	260	547	191	182	266	301	8703	**8703**
Total		1898			1923			377			2758			998			749				
**Adaptation options**																					
**No**	**No**	**153**	**160**	**162**	**226**	**374**	**295**	**11**	**25**	**26**	**216**	**388**	**320**	**106**	**275**	**86**	**139**	**198**	**216**	**3376**	**3376**
Yes	Combined	277	273	278	243	245	253	95	95	104	388	391	391	62	189	35	22	52	50	3443	5327
	Cultivar	4	1	4	2	48	46		1		6	50	86		4		2	1	10	265	
	Fertilizer			2	5	19	13			1	85	87	88	2	2	2		1	19	326	
	Irrigation	184	184	184	5	6	4				41	41	39	13	18	7	10	6	2	744	
	Planting date	6	2	6	16	64	27			3	25	57	29		8		9	5	3	260	
	Soil organic matter management			6			6						6							18	
	Tillage						4			4										8	
	Others		8	4	3	12	7		8	4	3	14	7	77	51	61		3	1	263	
CO2	Without CO2 fertilisation effect	9	9	12	14	49	44		8	9	12	54	53	7	12	8	31	36	44	411	**8703**
	With CO2 fertilisation effect	615	619	634	486	719	611	106	121	133	752	974	913	253	535	183	151	230	257	8292	

NF: Near future (<2040)

MC:Mid centy (2040 = <2070)

EC: End century (2070 = <2100)

**Online-only Table 3:** Medians of the projected impacts on four major crops.

Crop	Region	RCP2.6			RCP4.5			RCP6.0			RCP8.5			SRES			Others			Sub total	Total
		NF	MC	EC	NF	MC	EC	NF	MC	EC	NF	MC	EC	NF	MC	EC	NF	MC	EC		
Maize	Africa	−1.7	−3.0	−4.9	−4.1	−9.9	−15.2	−1.1	0.0	−4.1	−2.7	−11.5	−21.1	−0.6	−15.3	−9.3	−4.0	−2.2	−13.3	−5.6	−2.89
	Asia	−2.3	−4.0	−4.9	−1.7	−3.8	−6.1	−0.6	0.3	−2.1	−1.5	−4.8	−18.2	−2.2	−8.3	−12.5	−4.5	−8.7	−13.2	−4.3	
	Central and South America	−1.6	−3.2	−4.4	−2.1	−6.6	−11.2	−0.9	−5.0	−5.8	−1.8	−7.6	−17.2	−0.9	−13.6	−4.5	−8.0	−10.1	−71.4	−4.8	
	Europe	1.1	2.8	2.6	0.8	1.5	1.9	0.3	1.2	2.0	1.1	−0.6	−8.3	−0.8	−10.0	−0.4	−4.3	−11.8	−13.8	0.1	
	North America	−0.9	0.2	0.2	−0.5	−7.6	−16.0	−4.0	−2.8	−16.8	−1.3	−7.0	−15.3	−0.2	−2.6	−28.2	−0.3	−6.0	0.7	−2.0	
	Global			−7.6		−5.6	−12.6			−15.5	−0.3	−0.6	−16.7							−5.3	
Rice	Africa	3.3	1.5	2.5	2.1	0.7	0.4				0.0	−0.4	−1.0	−1.0	−10.4	−7.2		−4.7		−0.5	−1.43
	Asia	−1.5	2.1	0.7	−1.3	−0.6	−1.3	1.0	7.4	5.0	−1.2	−2.5	−12.8	−0.9	−0.7	−2.4	−1.5	−7.8	−3.1	−1.5	
	Central and South America	5.1	6.9	6.0	6.1	9.2	12.5				0.7	−1.6	−9.3	−2.1	−6.0	2.3				3.2	
	Europe	9.3	0.9	3.4		9.4	−0.5				8.5	8.9	−4.6							1.1	
	North America	−4.9	−0.6	−4.4	−3.0	−2.0	−6.6				−10.6	−30.4	−46.8	0.3	−3.0	0.3				−6.5	
	Global			−2.9	2.0		−4.9			−6.0			−9.6							−4.9	
Soybean	Asia	−13.5	−13.7	−13.3	−14.6	−12.6	−19.0				−5.4	−11.4	−22.6	4.9	−12.9		−2.0	−2.7	0.0	−12.4	−12.00
	Central and South America	−40.0	−42.9	−44.6	−27.0	7.8	−32.1				−42.8	−40.8	−55.9	4.6	−13.4		−12.0	7.1	−71.4	−21.5	
	North America	−4.4	−12.9	−20.9	−17.3	−4.9	−11.6				−21.6	−3.7	−28.0	−4.2	−6.0	−17.7	1.7	−14.8	7.5	−4.5	
	Global			−3.2		9.0	−5.2			−6.4			−10.3							−5.2	
Wheat	Africa	−30.5	−28.0	−24.1	−30.0	−13.2	−15.6				−3.1	−5.7	−9.7	0.2	−12.5	−5.6		−2.9	−22.7	−13.6	−3.13
	Asia	−11.2	2.2	−1.7	−2.7	−2.9	−6.2	−4.8	12.6	15.3	−3.5	−4.3	−9.1	−0.6	−2.8	1.5	−4.7	−12.8	−16.0	−2.9	
	Australasia	−14.8	−21.8	−24.5	−22.6	−23.0	−35.6				−9.8	−17.7	−46.2	−1.2	−9.3	3.2	−3.3	−13.9	−27.8	−14.0	
	Central and South America	−13.9	−11.5	−10.0	−9.7	−10.6	−8.7				1.5	−2.9	−2.7	0.0	−6.8	4.6	1.0	−5.9	−3.0	−4.8	
	Europe	−4.1	−11.5	−5.5	−4.1	3.5	5.4			30.4	−4.4	5.0	3.5	1.4	−1.9	3.4	3.2	−0.1	−3.3	3.1	
	North America	−23.6	−26.0	−25.4	−23.0	−3.3	−1.6				−14.9	−3.0	−14.3	1.3	−11.5	−14.5	−2.4	−0.2	−10.2	−2.8	
	Global		1.6	−6.1		1.7	−3.6			−12.4		2.3	−19.9							−2.3	
Sub total		−1.4	−1.3	−2.0	−1.9	−2.9	−3.9	−0.9	1.1	−3.0	−1.3	−3.9	−11.8	−1.0	−7.4	−3.9	−2.0	−3.9	−5.8		−**2.78**
Total				−1.5			−2.8			−0.3			−4.1			−3.5			−3.2		
**Adaptation options**																					
No	No	−9.0	−7.9	−7.7	−4.0	−4.7	−7.0	−0.9	8.3	−6.2	−5.7	−6.2	−18.0	−0.9	−12.8	−7.7	−1.3	−4.5	−6.0	−6.2	−6.22
Yes	Combined	−1.2	−2.3	−2.5	−1.1	−3.2	−4.3	−0.8	−0.3	−2.1	−1.1	−5.0	−12.9	−1.7	−4.9	−6.7	−2.0	−2.7	−7.9	−2.7	−1.64
	Cultivar	20.7	−2.6	24.5	4.6	3.7	5.6		−7.8		18.9	5.7	5.6		5.0		−4.3	3.1	−5.8	5.0	
	Fertilizer			6.6	6.5	2.1	−0.7			17.3	0.6	0.0	−5.4	−2.1	−10.7	−18.5		9.0	−1.5	−0.6	
	Irrigation	1.0	1.7	2.1	−2.9	−8.8	−23.1				1.1	−3.8	−9.0	−1.2	−1.8	−1.5	−3.3	1.4	22.1	0.2	
	Planting date	−4.0	1.3	−4.3	−2.4	−1.7	−4.4			−24.9	−2.2	−3.7	−13.6		−7.1		−2.9	−12.0	5.1	−3.8	
	Soil organic matter management			6.0			−0.2						1.3							1.8	
	Tillage						−25.1			−19.5										−21.9	
	Others		16.8	17.0	−5.4	10.8	11.4		17.4	26.4	−6.6	14.3	14.3	−0.6	−1.4	−1.0		−8.6	−5.6	−0.5	
CO2	Without CO2 fertilisation effect	−7.5	10.1	1.0	−2.8	−5.6	−8.7		10.5	−6.0	−2.8	−6.0	−15.4	−4.2	−1.9	−24.7	−2.1	−8.9	−16.2	−6.1	−**2.78**
	With CO2 fertilisation effect	−1.2	−1.4	−2.0	−1.8	−2.8	−3.7	−0.9	0.3	−2.7	−1.3	−3.8	−11.5	−0.9	−7.7	−3.8	−1.8	−2.9	−3.6	−2.6	

NF: Near future (<2040)

MC:Mid centy (2040 = <2070)

EC: End century (2070 = <2100)

**Online-only Table 4:** Means of the projected impacts on four major crops.

Crop	Region	RCP2.6			RCP4.5			RCP6.0			RCP8.5			SRES			Others			Sub total	Total
		NF	MC	EC	NF	MC	EC	NF	MC	EC	NF	MC	EC	NF	MC	EC	NF	MC	EC		
Maize	Africa	−6.9	−6.5	−7.1	−15.7	−13.2	−20.8	−1.3	−1.7	−6.3	−9.3	−14.0	−26.0	−0.5	−14.4	−7.1	−2.2	−6.6	−17.4	−11.3	−8.0
	Asia	−9.2	−9.4	−9.1	−15.1	−10.7	−19.8	−0.4	0.4	−2.8	−10.0	−11.1	−24.7	−2.2	−8.2	−10.5	−5.6	−13.1	−19.2	−11.0	
	Central and South America	−8.1	−7.6	−7.7	−19.9	−22.2	−27.6	−0.5	−3.7	−7.1	−11.5	−16.3	−27.1	−1.1	−13.5	−3.7	−10.3	−9.4	−59.5	−13.9	
	Europe	0.6	2.3	3.4	0.2	0.1	−0.7	0.2	1.1	−0.3	0.5	−1.5	−10.5	−1.9	−6.5	−3.6	−6.7	−22.6	−19.8	−1.4	
	North America	−2.4	0.1	−0.6	−2.4	−5.8	−8.3	−2.7	−1.6	−7.2	−1.5	−7.6	−20.7	−1.8	−4.8	−24.0	1.8	−5.8	4.0	−4.4	
	Global			−7.6		−5.6	−12.6			−15.5	0.2	−1.8	−17.9							−6.0	
Rice	Africa	−1.6	−1.7	−2.5	−2.1	−0.9	−0.4				−1.9	−1.7	−4.6	−1.8	−8.4	−6.9		−7.5		−3.6	−3.4
	Asia	−5.3	−3.3	−2.3	−2.5	−1.7	−2.3	−11.0	−5.9	−1.8	−2.6	−4.0	−11.2	−0.7	−1.4	−1.1	−1.8	−8.7	−3.4	−3.8	
	Central and South America	5.1	6.9	6.0	6.1	9.2	12.5				2.3	2.5	−1.8	−2.1	−6.0	2.3				3.4	
	Europe	8.3	0.9	9.3		9.4	−0.5				7.5	8.9	−2.2							5.5	
	North America	−4.9	−0.6	−4.4	−3.0	−2.0	−6.6				−10.4	−28.4	−42.2	0.3	−3.0	0.3				−13.7	
	Global			−2.9	2.0		−4.9			−6.0			−9.6							−4.3	
Soybean	Asia	−27.0	−27.2	−31.1	−29.4	−29.5	−36.9				−14.4	−26.0	−34.8	4.9	−13.1		−2.5	−5.4	−8.9	−23.0	−15.6
	Central and South America	−36.4	−34.8	−39.2	−22.8	−6.6	−13.7				−40.8	−15.2	−18.9	4.6	−13.5		−9.7	4.7	−60.1	−17.8	
	North America	−4.5	−9.6	−20.9	−17.3	−5.2	−15.3				−21.6	−8.1	−32.9	−4.2	−3.1	−17.7	1.7	−14.8	5.4	−5.7	
	Global			−3.2		9.0	−5.2			−6.4			−10.3							−3.2	
Wheat	Africa	−37.3	−35.4	−30.4	−38.2	−23.2	−22.5				−20.7	−15.9	−15.1	0.2	−10.8	−5.6		0.0	−10.3	−19.7	−6.1
	Asia	−14.7	−3.9	−7.6	−4.2	−3.7	−8.9	−4.8	15.2	16.7	−7.5	−4.5	−10.3	0.1	−5.1	4.5	−4.9	−12.8	−17.0	−5.7	
	Australasia	−23.6	−26.0	−25.4	−23.0	−4.3	−5.4				−10.9	−5.2	−12.7	1.3	−11.3	−12.9	−2.4	2.1	−3.4	−6.8	
	Central and South America	−25.5	−32.3	−30.3	−30.3	−32.7	−40.4				−18.4	−24.7	−−39.0	−0.5	−9.9	0.5	−3.3	−−13.3	−27.8	−22.3	
	Europe	−10.7	−8.9	−7.7	−9.8	−7.0	−5.9				−3.8	−1.3	−0.5	−0.5	−7.2	2.5	−0.4	−6.5	−4.3	−5.1	
	North America	−8.9	−11.4	−3.2	−9.4	2.8	6.5			29.7	−7.1	3.3	4.0	1.4	2.6	2.7	9.4	−0.3	3.2	3.2	
	Global		1.6	−6.1		1.7	−3.6			−12.4		0.4	−19.9							−4.1	
Sub total		−6.7	−5.8	−5.3	−8.9	−6.2	−8.7	−1.4	1.3	−1.7	−6.0	−7.0	−13.9	−1.1	−7.5	−4.9	−0.8	−5.0	−7.9		−**6.98**
Total				−5.9			−7.8			−0.6			−9.1			−5.3			−5.1		
Adaptation options																					
No	No	−16.8	−15.1	−14.8	−10.8	−7.7	−11.6	−9.4	2.4	−3.4	−12.5	−10.5	−21.1	−0.9	−10.9	−7.8	−0.2	−5.1	−8.3	−10.8	−10.8
Yes	Combined	−7.5	−7.6	−7.2	−8.3	−8.9	−8.9	−0.5	−0.4	−1.8	−5.1	−8.5	−16.1	−1.6	−4.9	−6.1	−3.2	−4.6	−12.7	−7.8	−4.6
	Cultivar	21.2	−2.6	25.0	4.6	3.7	6.6		−7.8		14.3	5.1	7.7		6.7		−4.3	3.1	−4.2	6.2	
	Fertiliser			6.6	6.7	8.2	−3.4			17.3	0.3	1.4	−4.7	−2.1	−10.7	−18.5		9.0	1.1	−0.3	
	Irrigation	2.1	4.0	4.5	1.3	−7.0	−17.8				2.2	−1.3	−6.8	0.2	−2.6	1.4	−3.3	1.7	22.1	2.2	
	Planting date	−2.5	1.3	−4.5	−1.1	−1.8	−6.5			−25.4	−2.7	−4.6	−12.7		−7.8		−0.3	−11.6	4.5	−4.7	
	Soil organic matter management			4.1			1.5						−0.6							1.7	
	Tillage						−8.4			−1.5										−4.9	
	Others		17.0	17.2	−5.6	7.7	−1.8		18.8	26.6	−7.0	10.3	2.1	−1.0	−2.0	−0.3		−12.8	−5.6	1.6	
CO2	Without CO2 fertilisation effect	−7.3	9.3	2.2	−3.0	−3.5	−6.2		10.4	0.1	−2.8	−4.7	−14.5	−3.8	−1.7	−23.9	−2.3	−8.4	−18.1	−6.9	−**6.98**
	With CO2 fertilisation effect	−6.7	−6.0	−5.4	−9.1	−6.4	−8.9	−1.4	0.7	−1.8	−6.0	−7.1	−13.9	−1.0	-7.7	−4.0	−0.4	−4.5	−6.2	−7.0	

NF: Near future (<2040)

MC:Mid centy (2040 = <2070)

EC: End century (2070 = <2100)
