# Supplementary material for: A global dataset for the projected impacts of climate change on four major crops
Source: Sci Data. 2022 Feb 16;9:58. doi: 10.1038/s41597-022-01150-7 (PMC8850443; doi:10.1038/s41597-022-01150-7)
Supplement: Supplementary file 1 — Supplementary materials [file 41597_2022_1150_MOESM1_ESM.pdf]

Data Descriptor – Supplementary Materials

Title

A global dataset for the projected impacts of climate change on four major crops

Authors

Toshihiro Hasegawa[1], Hitomi Wakatsuki[1], Hui Ju[2], Shalika Vyas[3], Gerald C. Nelson[4], Aidan Farrell[5], Delphine Deryng[6], Francisco Meza[7], David Makowski[8]

Affiliations

1. Institute for Agro-Environmental Sciences, National Agricultural and Food Research Organization, Tsukuba, Ibaraki, 305-8604, Japan
2. Institute of Environment and sustainable Development in Agriculture, Chinese Academy of Agricultural Sciences (IEDA,CAAS), Beijing, 100081, China
3. Borlaug Institute for South Asia, CIMMYT, New Delhi, 110012, India
4. University of Illinois, Urbana, IL.,USA
5. The University of the West Indies, St. Augustine, Trinidad
6. IRI THESys, Humboldt-Universität zu Berlin, Berlin, 10099, Germany
7. Pontificia Universidad Católica de Chile, Santiago, Chile
8. Applied mathematics and computer science (MIA 518), INRAE AgroParisTech, Université Paris-Saclay, 75231, Paris, France.

corresponding author(s): Toshihiro Hasegawa (thase@affrc.go.jp)

Supplementary Table S1. Comparison of four random forest models with different combinations of for estimating local temperature rise

Model	1	2	3	4
# of variables used	3	3	5	4
Importance of variables				
Global temperature rise	1.829	1.090	0.934	1.698
Longitude	0.290		0.148	0.266
Latitude	0.359		0.197	0.390
RCP		0.404	0.394	
Future Midpoint year		0.491	0.488	
Current average temperature				0.329
Fit MSE	0.031	0.133	0.051	0.043
Fit percent variance explained	97.14	87.71	95.41	95.99
Median permuted MSE	0.035	0.135	0.053	0.047
Median permuted percent variance explained	96.79	87.55	95.09	95.66
Median cross-validation RMSE	0.183	0.367	0.195	0.215

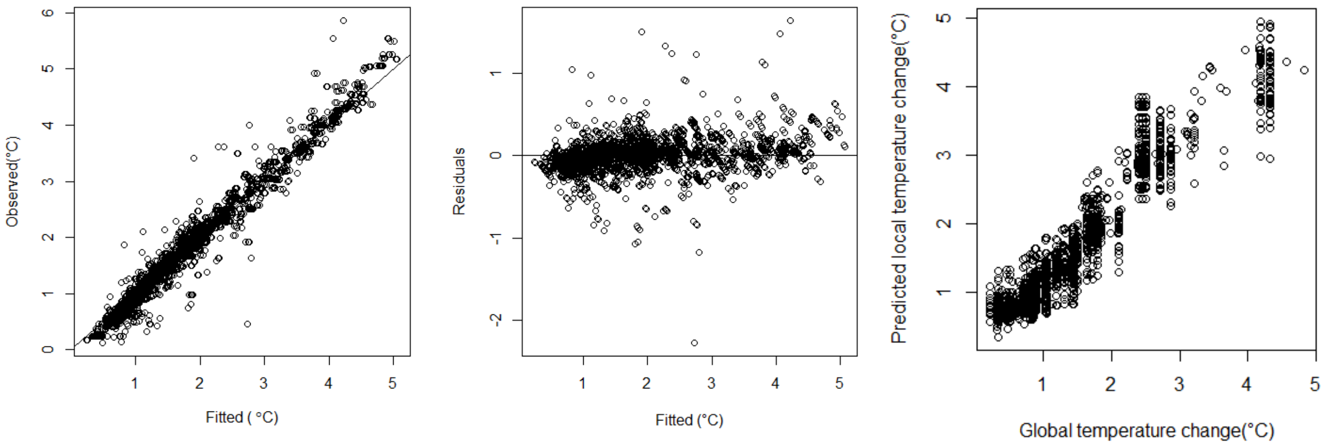

Supplementary Fig. S1. Goodness of fit for the Random Forest model for estimating local temperature rise as a function of global temperature rise, latitude and longitude (Model 1 in Table S1). (a) Observed and fitted, (b) Residual plot, (c) Cross-validation prediction

Supplementary Table S2. Comparison of four random forest models with different combinations of six variables for estimating global temperature

Model	1	2	3	4
# of variables used	3	3	5	4
Importance of variables				
Global temperature rise	1.226	0.346	0.443	1.164
Longitude	0.183		0.065	0.161
Latitude	0.226		0.071	0.251
RCP		0.405	0.310	
Future Mid-point year		0.453	0.384	
Current average temperature				0.194
Fit MSE	0.025	0.021	0.015	0.032
Fit percent variance explained	96.18	96.77	97.84	95.11
Median permuted MSE	0.028	0.022	0.016	0.036
Median permuted percent variance explained	95.74	96.56	97.59	94.54
Median cross-validation RMSE	0.16016	0.14452	0.12132	0.18314

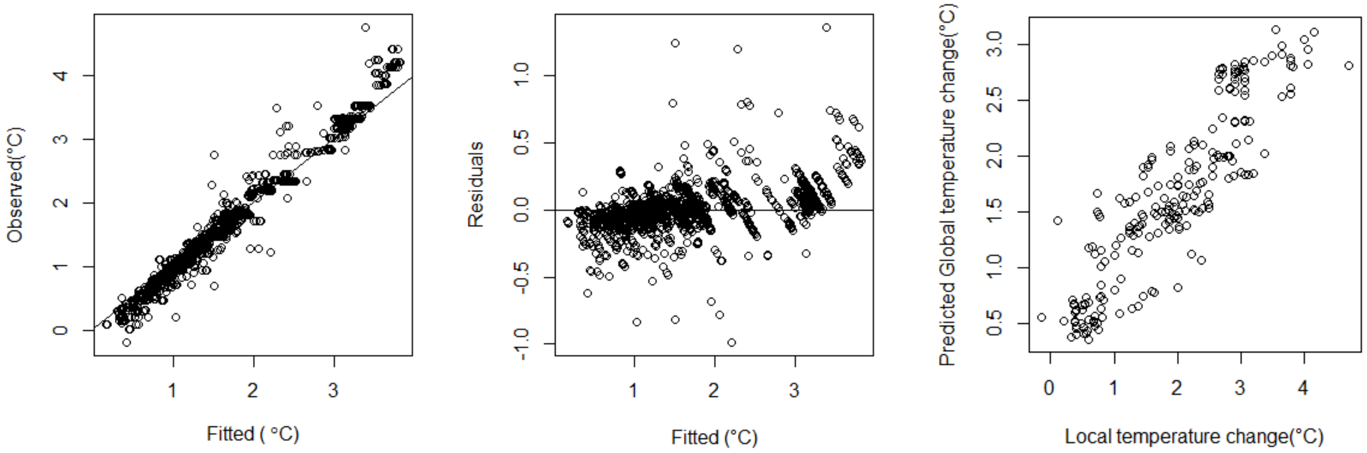

Supplementary Fig. S2. Goodness of fit for the Random Forest model for estimating global temperature rise as a function of local temperature rise, latitude and longitude (Model 3 in Table S2). (a) Observed and fitted, (b) Residual plot, (c) Cross-validation prediction

Supplementary Table S3. Comparison of eight random forest models with different combinations of ten variables for estimating global temperature

Model	1	2	3	4	5	6	7	8
# of Variables used	4	4	3	7	5	8	5	10
Importance of variables								
Delta_temp_global	19737	38621	24887	4601	16785		13112	2823
Dleta_temp_local	25213	39903	30632	10421	21922	8438	16885	6610
Longitude	8969			6570	7608	6682	8254	6166
Latitude	7888			3730	6270	3571	8719	2788
RCP		16192		2047	5366	2511		1104
FMP		31496		4985		4682		3093
Pr_annual			11933	7860		7577		6375
Tave							8106	3837
Species								2473
Effect								2050
Fit MSE	621	2753	800	300	820	399	763	416
Fit percent variance explained	93.60	71.26	91.91	96.89	91.36	95.82	91.95	95.82
Median permuted MSE	708	2850	919	365	938	923	814	464
Median permuted percent variance explained	92.49	69.80	90.35	96.14	90.10	89.95	91.37	95.08
Median cross-validation RMSE	25.4	52.8	29.5	18.2	28.6	30.1	28.4	20.7

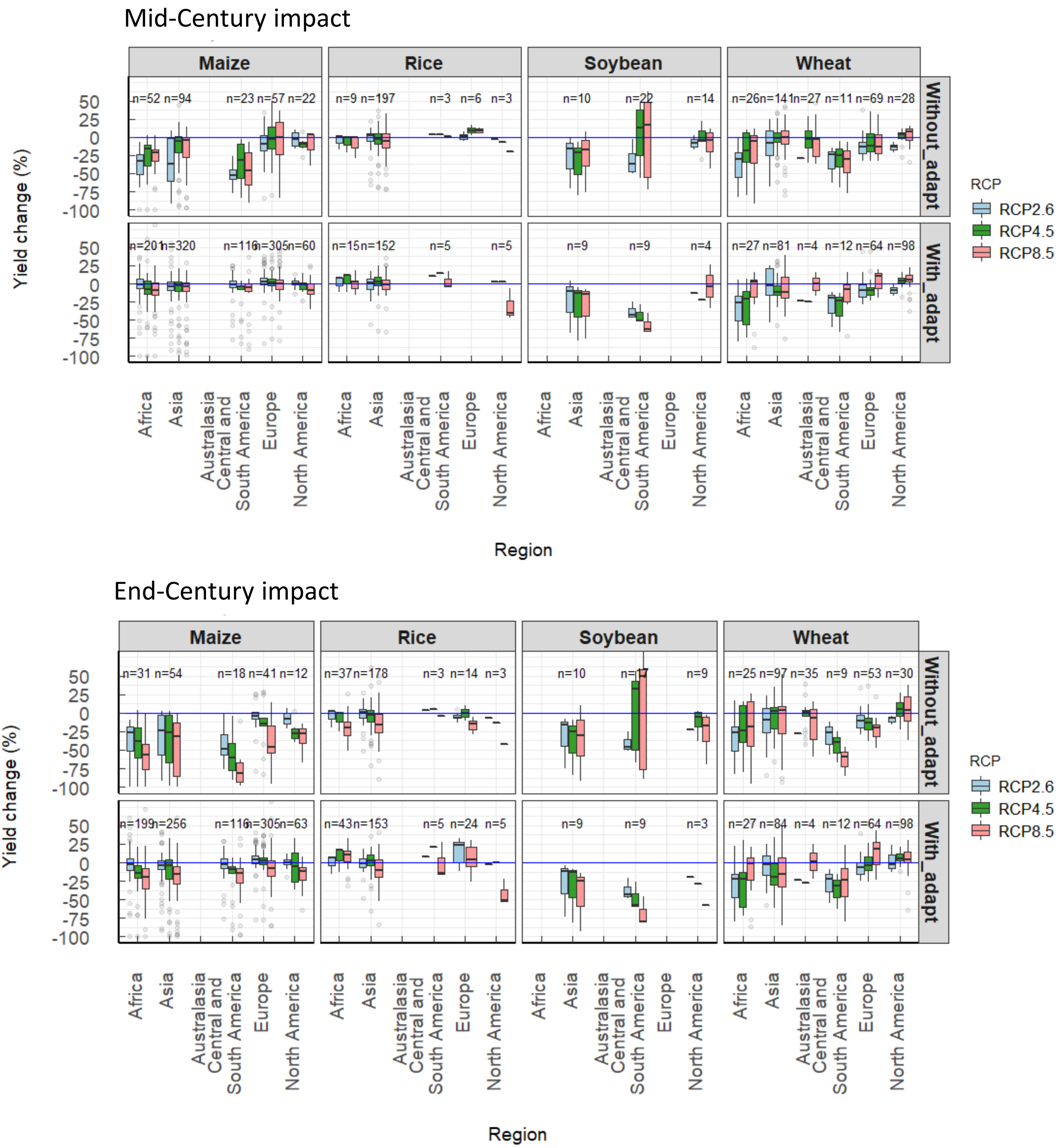

Supplementary Fig. S3 Climate change impacts on four crops in the mid 21st century with and without adaptation in IPCC regions by regions at mid-century (MC, 2040-2069, upper panels) and end-century (EC, 2070-2100, lower panels). n is the number of simulations. The box is the interquartile range (IQR) and the middle line in the box represents the median. The upper- and lower-end of whiskers are median 1.5 $\times$ IQR $\pm$ median. Open circles are values outside the 1.5 $\times$ IQR.

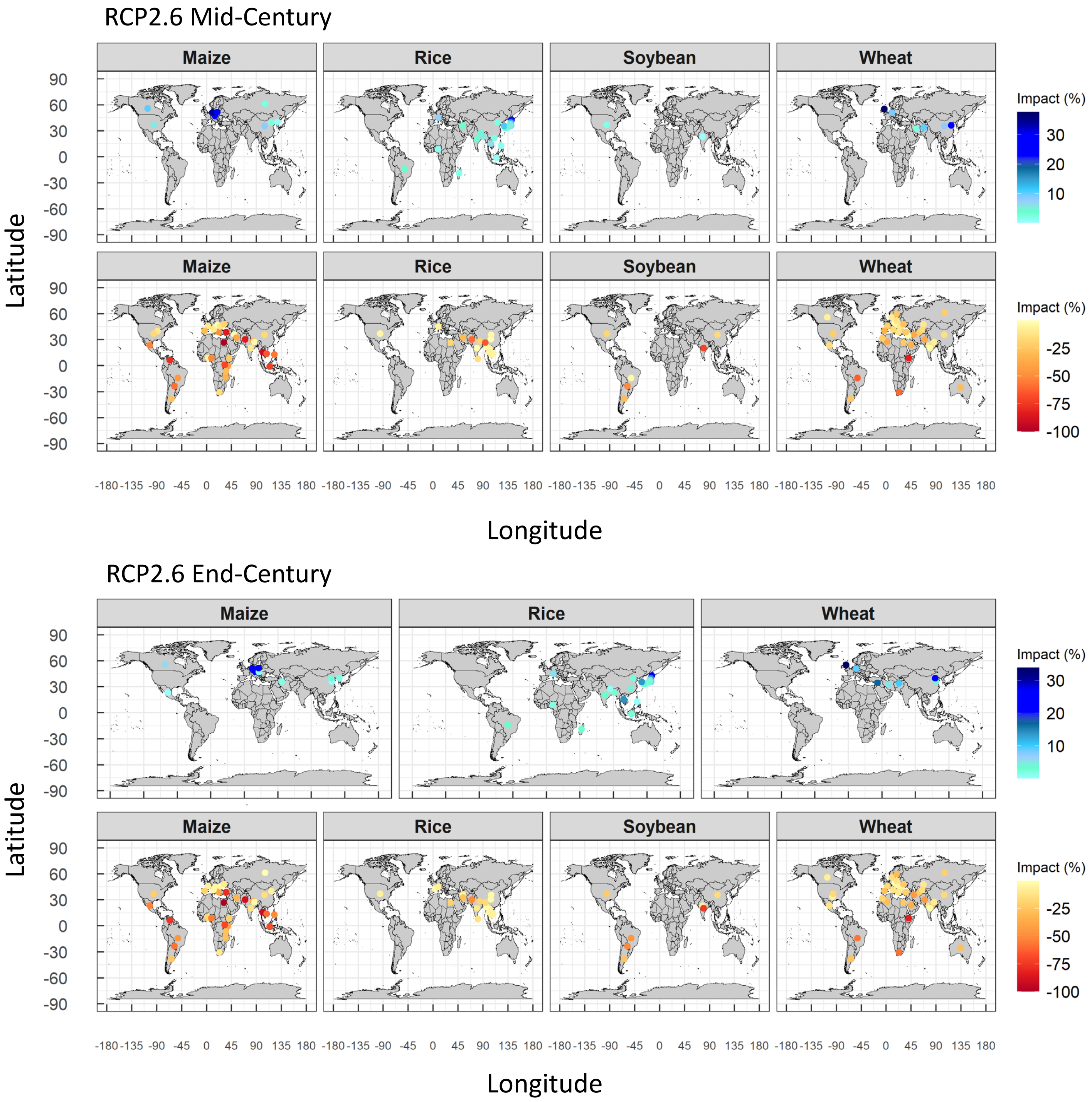

Supplementary Figure S4(a) Climate change impacts on four crops without adaptation under RCP2.6.

Upper two panels, Mid-century; Lower two panels, End-Century.

Maps with bluish symbols, positive effects; Maps with reddish symbols, negative effects.

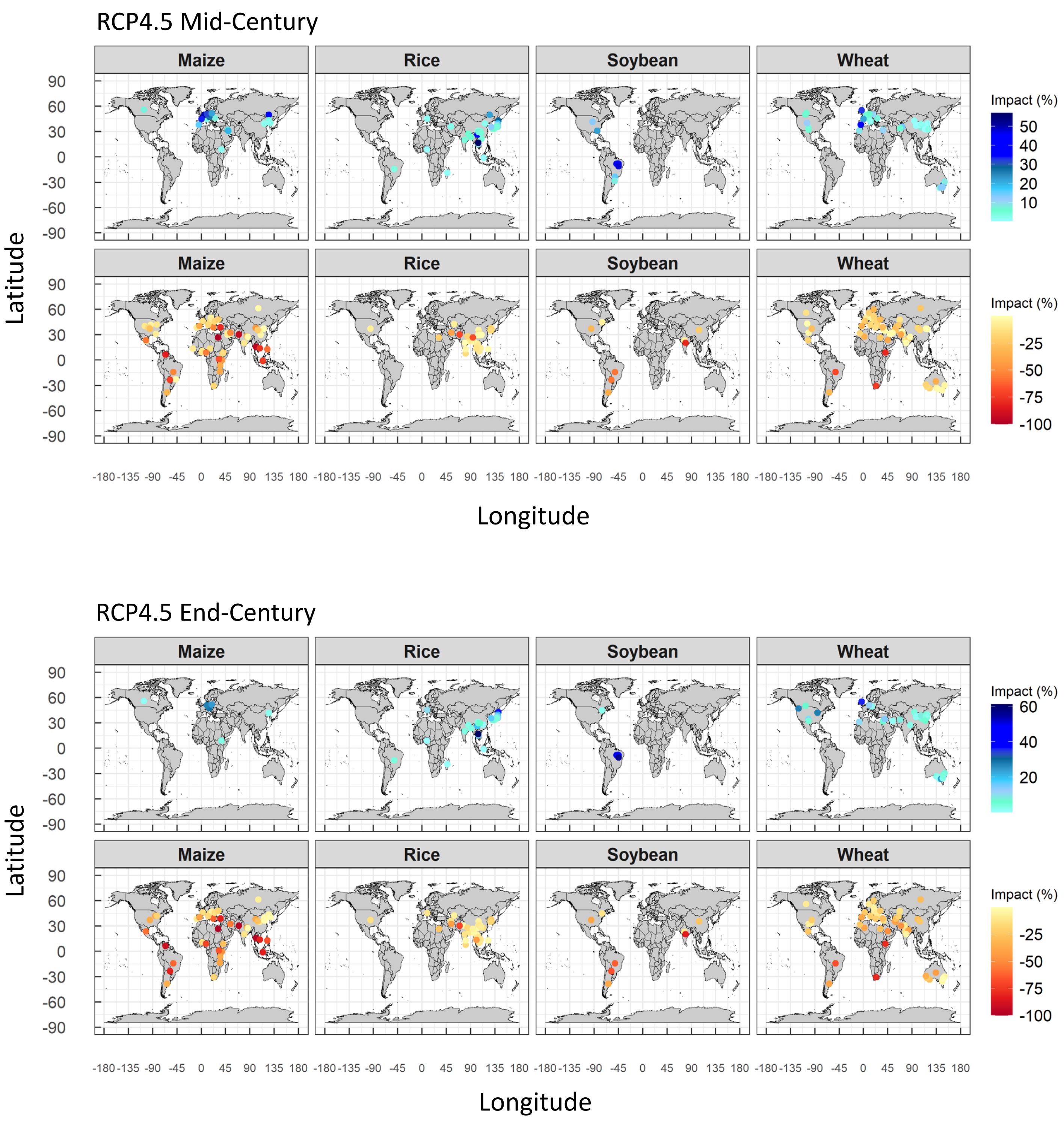

Supplementary Figure S4(b) Climate change impacts on four crops without adaptation under RCP4.5.
Upper two panels, Mid-century; Lower two panels, End-Century.
Maps with bluish symbols, positive effects; Maps with reddish symbols, negative effects.

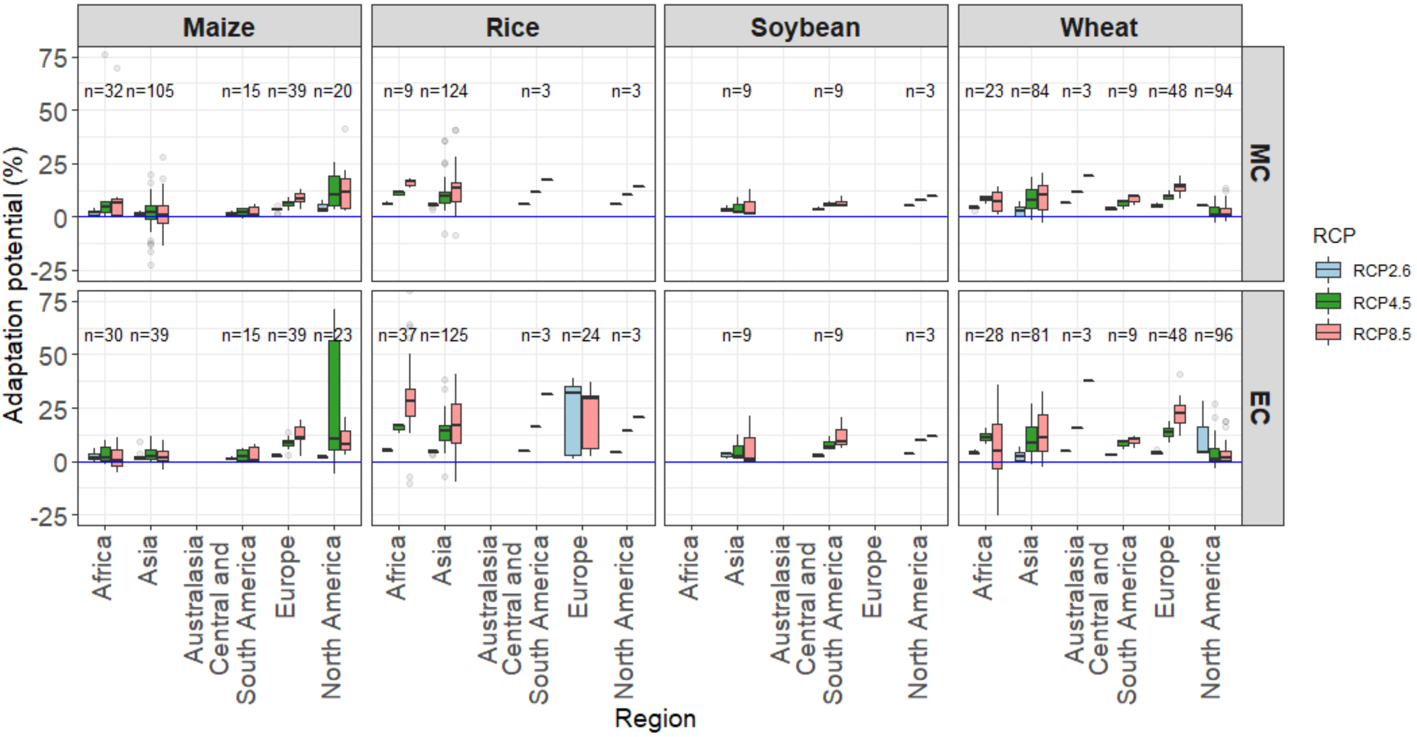

Supplementary Fig.S5 Adaptation potential, defined as the difference between yield impacts with and without adaptation in projected impacts by regions at mid-century (MC, 2040-2069, upper panels) and end-century (EC, 2070-2100, lower panels). n is the number of simulations. The box is the interquartile range (IQR) and the middle line in the box represents the median. The upper- and lower-end of whiskers are median 1.5 × IQR ± median. Open circles are values outside the 1.5 × IQR.
